# CheS-Mapper 2.0 for visual validation of (Q)SAR models

**DOI:** 10.1186/s13321-014-0041-7

**Published:** 2014-09-23

**Authors:** Martin Gütlein, Andreas Karwath, Stefan Kramer

**Affiliations:** 1 Institute for Physics, Albert-Ludwigs-Universität Freiburg, Hermann Herder Str. 3, Freiburg D-79104, Germany; 2 Information Systems, Institut für Informatik, Johannes Gutenberg - Universität Mainz, Staudingerweg 9, Mainz D-55128, Germany

**Keywords:** Visualization, Validation, (Q)SAR, 3D space

## Abstract

**Background:**

Sound statistical validation is important to evaluate and compare the overall performance of (Q)SAR models. However, classical validation does not support the user in better understanding the properties of the model or the underlying data. Even though, a number of visualization tools for analyzing (Q)SAR information in small molecule datasets exist, integrated visualization methods that allow the investigation of model validation results are still lacking.

**Results:**

We propose visual validation, as an approach for the graphical inspection of (Q)SAR model validation results. The approach applies the 3D viewer CheS-Mapper, an open-source application for the exploration of small molecules in virtual 3D space. The present work describes the new functionalities in CheS-Mapper 2.0, that facilitate the analysis of (Q)SAR information and allows the visual validation of (Q)SAR models. The tool enables the comparison of model predictions to the actual activity in feature space. The approach is generic: It is model-independent and can handle physico-chemical and structural input features as well as quantitative and qualitative endpoints.

**Conclusions:**

Visual validation with CheS-Mapper enables analyzing (Q)SAR information in the data and indicates how this information is employed by the (Q)SAR model. It reveals, if the endpoint is modeled too specific or too generic and highlights common properties of misclassified compounds. Moreover, the researcher can use CheS-Mapper to inspect how the (Q)SAR model predicts activity cliffs. The CheS-Mapper software is freely available at http://ches-mapper.org.

**Graphical abstract:**

Comparing actual and predicted activity values with CheS-Mapper.

## 1 Background

Visualization of (quantitative) structure-activity relationship ((Q)SAR) information in chemical datasets is a very active field of research in cheminformatics [1]-[8]. Many approaches are being developed that help to understand existing correlations between the structure of chemical compounds, their physico-chemical properties, and biological or toxic effects. These correlations are employed by (Q)SAR models to predict the activity of unseen compounds. The predictive performance of (Q)SAR models can then be evaluated with numerous statistical validation techniques. There is however, to the best of the authors’ knowledge, no visualization method yet that incorporates (Q)SAR predictions and validation results. One reason for this might be that most (Q)SAR models are the results of applying statistical machine learning approaches to chemical datasets and the resulting models are sometimes opaque and it is commonly not an easy task to extract the reasoning behind a prediction. Some models induced by machine learning approaches, however, are relatively easy to understand, like decision trees, rule learners or nearest neighbor models. The predictions of these models are easy to comprehend, as long as the number of features that are employed for predictions is not too large (e.g. the size of the decision tree is reasonably small). Therefore, several model-dependent visualization tools exist [9]-[12]. In contrast, many other models can rather be seen as black boxes, like artificial neural networks or support vector machines. In addition, these complex models are often more predictive than intuitive and simpler models.

**Figure 1:**
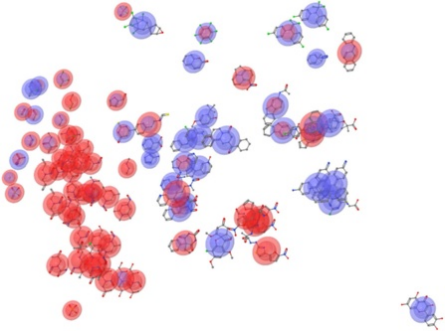
**Comparing actual and predicted activity values with CheS-Mapper.** The CPDB hamster dataset is embedded into 3D space, based on 14 physico-chemical (PC) descriptors that have been computed with CheS-Mapper using Open Babel. The compounds are predicted with a 3-Nearest Neighbor approach employing the same PC features. The inner sphere corresponds to the actual endpoint activity, with class values active (red) and inactive (blue). The outer, flattened spheroid depicts the prediction.

In this paper, we propose a model-independent visual analysis of validation results employing the 3D viewer CheS-Mapper [13]. The presented approach does not investigate how predictions are made by each model, but rather allows the comparison of actual and predicted activity values in the feature space (see Figure 1). We call this approach *visual validation*. It should be regarded as complementary to the standard statistical validation. Visually examining (Q)SAR model validation results can aid in understanding the model itself as well as the modeled data, and can furthermore yield the following benefits:

**Data curation:** It is important to inspect (groups of) misclassified compounds (in case of classification), or compounds with high prediction error (regression). Investigating possible reasons for the erroneous predictions might aid in detecting errors in the training data, like mis-measured endpoint values. The researcher might as well discover that the misclassifications are outliers or that more training data is required.

**Model improvement:** Another possible reason for bad model performance may be improper feature choice, e.g. the available features can not be used to distinguish between some active and inactive compounds. Moreover, the selected model might be too specific (overfitting) or too general (underfitting). Additionally, visual validation can show the effect of different model parameters.

**Mechanistic interpretation:** It is also possible to extract knowledge from groups of compounds that are correctly classified. Compounds with similar feature values and endpoint values might have similar modes of action. Consequently, visual validation can support the researcher in deriving a mechanistic interpretation. Mechanistic interpretation and proper model validation are requirements of the OECD guidelines for valid (Q)SAR models [14]. To this end, visual validation could also help to improve the acceptance of (Q)SAR models by regulatory authorities as alternative testing methods.

### 1.1 Existing visualization tools for small molecule datasets

The most important requirement for visualization approaches is to help detecting correlations in multivariate and structure data. One possible method to analyze correlations is to apply dimensionality reduction. Scatter plots can visualize how and if the endpoint values are correlated to the selected properties or features, but are limited to two or three dimensions. Hence, dimensionality reduction is applied to transform the multi-dimensional feature space to two or three numeric features while trying to preserve the data structure [15]. Various variants of dimensionality reduction are applied in cheminformatics [16],[17] and many visualization tools for small molecule datasets include these dimensionality reduction techniques (ChemSpaceShuttle [18], MQN-Mapplet [2], Screening Assistant 2 [3], ViFrame [4], HiTSEE KNIME [5]).

Other visualization tools rely on clustering (Molecular Property eXplorer [19], Radial Clustergrams [20]). Clustering compounds into subgroups according to their properties can provide useful information: clusters might resemble chemical categories having similar properties or sharing a common activity profile.

In general, tree-based or graph-based approaches convert the dataset into connected data structures (LASSO graph [6], SALI networks [21], SARANEA [22], Scaffold Hunter [7], Similarity–Potency Trees [23]). Hence, nodes in the trees (or graphs) correspond to compounds and/or groups of compounds. The proximity of the nodes reflects the (dis-)similarity of the employed feature values. These tools can usually highlight the activity value of compounds, and are therefore suitable for (Q)SAR information analysis as well.

(Q)SAR information in chemical datasets is usually hard to comprehend. The (Q)SAR assumption is that compounds with similar structure tend to have similar chemical and biological properties [24]. Consequently, small changes in structure often cause only small changes in activity. In this case, the so-called *activity landscape*[25] is considered to be smooth. The term activity landscape describes the distribution of endpoint values in the feature space. However, sometimes small changes in structure can cause big changes in activity. This is referred to as *activity cliff*[26],[27]. Therefore, an activity cliff can be defined by two compounds that have very similar feature values, but largely differing endpoint values. Activity cliffs are often visualized with the already mentioned approaches for visualization. Moreover, heat-maps (matrices of colored cells) are employed to highlight the corresponding pairs of compounds (Toxmatch 2 [8],[21]).

### 1.2 Existing visualization approaches for model validation

As mentioned above, approaches for the graphical analysis of machine learning models are often model dependent [10],[12],[28]. An example can be seen in an interactive visualization approach displaying decision trees using bars for each tree node [9]. Each bar contains colored instances, sorted according to the corresponding feature that is employed in this node. The coloring reflects the class distribution. Split points are indicated by lines, and can be modified or removed by the user. The system then rebuilds the tree according to the manualmodifications.

Mineset is a data-mining tool [11] that allows to create various machine learning models and provides different visualization approaches. This includes 3D scatter plots, as well as model-dependent views for decision tree or Naive Bayes models. However, the software is currently not available (according to email correspondence, the distributing company is planning to release a re-engineered beta version in 2014).

Another approach for model independent visualization of classification results uses a 2D projection of the predicted dataset with self-organizing maps (SOMs) [29]. Empty regions in the feature space are filled by sampling new instances. The maps are colored according to the class probability that is provided by the model for each prediction. The decision border (50% class probability) is indicated with a white line. Feature contours can be drawn over the map in order to interpret the space. Moreover, test instances can be overlayed, with their actual class colored, to show misclassified instances.

A model independent method is especially aimed for multi-class problems (classification with more than two classes) [30]. The visualization is exclusively based on the probability estimate provided by the classifier for each class value. The resulting plot displays a circle that is divided into radiants, each radiant accounts for one class. The more confident the classifier is with the prediction, the closer this instance is drawn to the edge of the circle in the corresponding radiant. However, this approach is limited, as it ignores the actual feature values of the instances itself.

None of the available cheminformatics visualization tools focuses on visualizing (Q)SAR model validation results as such. The authors of ChemSpaceShuttle [18] discuss how their tool can be used to embed compounds with two different class values (drug/non-drug) into 3D space. Different embeddings based on different sets of feature values were investigated to decide which feature set is most suitable for separating the compounds according to their class values. However, this work did not include (Q)SAR modeling. Moreover, the software neither draws compound structures nor computes compound feature values.

### 1.3 The 3D viewer CheS-Mapper

In this work, we present new developments of our visualization tool CheS-Mapper (Chemical Space Mapper) [13], specifically aimed towards visual validation. CheS-Mapper is a general, interactive, and open-source software that can be employed to inspect chemical datasets of small molecules. It maps the compounds into virtual 3D space and was designed to enable scientific researchers to investigate compounds and their features. Compared to existing methods, CheS-Mapper is a unique combination of clustering, dimensionality reduction, and 3D viewer. The distinguishing feature of the tool is that each compound is represented by its (3D) structure instead of substituting it by a dot or a node. In contrast to some existing open-source tools that are limited to a distinct operating system, depend upon the installation of an additional database or require a specific input format, CheS-Mapper is platform independent, requires no installation, and accepts a wide range of chemical formats.

The workflow of the software is divided into a data preprocessing part and a visualization part. We refer to the first part as Chemical Space Mapping. It can be configured with a dedicated graphical wizard that guides through the preprocessing steps. After loading the chemical dataset into the application, 3D structures for compounds can be computed if not already available. Subsequently, the user can select the compound features that are employed within the mapping process. Consequently, the compounds are grouped together into clusters and embedded into 3D space based on a user-defined chemical or biological similarity. A range of chemical descriptors and structural features can be computed by CheS-Mapper and numerous clustering and embedding algorithms are available. Finally, the compounds of each cluster can be aligned in 3D space according to common substructures. The second part of the application, the visualization, is based on a molecular 3D viewer and allows to explore the dataset in virtual 3D space. The user can rotate the dataset or zoom in on single compounds. The coloring of compounds can be adjusted to highlight cluster assignments, compound features, endpoint values, and structural fragments. Furthermore, the 3D aligned compounds of each cluster can be superimposed to highlight structural (dis-)similarities.

## 2 Implementation

The CheS-Mapper software is implemented in Java. It is provided as a Java Web Start application, which can directly be started from a web browser. Additionally, the program can also be downloaded as stand-alone version. CheS-Mapper is an open-source project hosted at GitHub (http://github.com/mguetlein/ches-mapper). The code architecture allows developers to easily integrate novel algorithms, e.g. for clustering, 3D structure calculation, etc. A range of Java libraries is integrated into the project: the 3D viewer for molecules Jmol [31], the Chemistry Development Kit (CDK [32]), and the data mining workbench WEKA [33]. Extended functions are provided in CheS-Mapper in case the free software tools Open Babel [34] and R [35] are installed on the local computer. Open Babel is a C++ library for cheminformatics that can be used for additional 3D computing, matching of SMARTS (Smiles Arbitrary Target Specification) fragments, and structural fragment mining. The statistical computing tool R is exploited by CheS-Mapper for clustering and embedding.

## 3 Visual validation of (Q)SAR models

The following section introduces new functionality of CheS-Mapper 2.0 for (Q)SAR information analysis and visual validation. Next, we describe how the tool can be used to visually validate (Q)SAR models. In the subsequent section, we present actual use cases and how the new features of CheS-Mapper 2.0 were employed to achieve the goals of the use cases. An overview of the new features of CheS-Mapper 2.0, the use cases, and the connection among them is shown in Table 1.

**Table 1:** Overview of new features and their application to a variety of use cases

	**New feature**						
(*a*)	Sorting of compound/cluster list according to selected feature						
(*b*)	Highlighting two features simultaneously						
(*c*)	Computing embedding quality and distances						
(*d*)	Determination of common properties of compounds/clusters						
(*e*)	Compute mean SALI values to detect activity cliffs						
(*f*)	KNIME integration						
**Dataset**	**Use case**	(*a*)	(*b*)	(*c*)	(*d*)	(*e*)	(*f*)
Caco-2	Inspect (Q)SAR information using integrated features	✓		✓		✓	
	Compare (Q)SAR models and validation methods	✓	✓		✓		✓
Cox-2	Inspect (Q)SAR information by mining structural fragments	✓		✓	✓	✓	
	Inspect validation results with respect to activity cliffs	✓			✓	✓	
EPA FHM	Inspect applicability domain algorithms	✓		✓	✓		
CPDB Hamster	Compare modeling with different feature sets	✓	✓	✓	✓	✓	
	(Provided in Additional file 2)						

### 3.1 New features for visual validation

**Figure 2:**
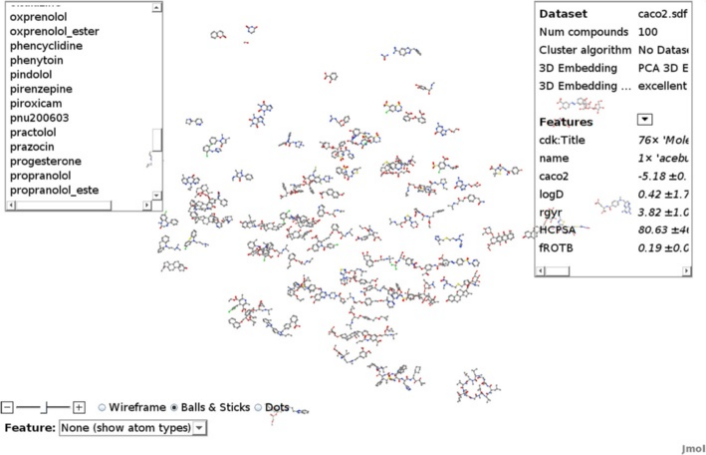
**The CheS-Mapper viewer showing the Caco-2 permeability dataset.** The compound list (on the left-hand side) can be used to select compounds. General dataset information and mean feature values are provided on the right-hand side. The control panel is located on the bottom left-hand side.

**Figure 3:**
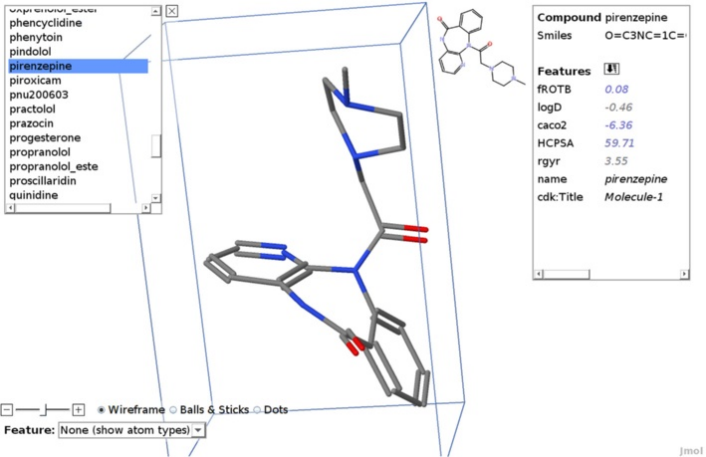
**Zooming in on compound*pirenzepine*.** The compound is depicted using the wireframe setting. Compound features are listed on the right-hand side. Feature values for *fROTB*, *caco2*, and *HCPSA* are relatively low and therefore colored in blue. The *fROTB* value of *pirenzepine* differs the most from the values in the entire dataset, therefore this feature is ranked at the top.

CheS-Mapper shows the compounds of the embedded dataset in the center of the 3D viewer (see Figure 2). The 3D positions of compounds have been calculated by the selected embedding algorithm. Hence, compounds that are similar based on the selected feature values, will be located close to each other in 3D space. When clicking on a compound, the view automatically zooms in on the structure (see Figure 3).

**Figure 4:**
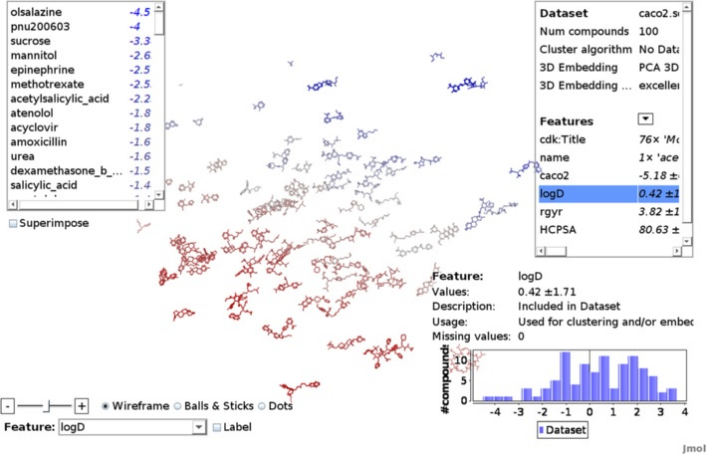
**Highlighting*logD* feature values in the Caco-2 permeability dataset.** The compound color has changed according to the feature value. Compounds with similar *logD* values are located close to each other in 3D space, as this feature was used for 3D embedding. The compound list at the left-hand side shows the *logD* value for each compound and the list is sorted according to the *logD* value. A histogram depicting the feature value distribution in the dataset is on the bottom right-hand side.

**Figure 5:**
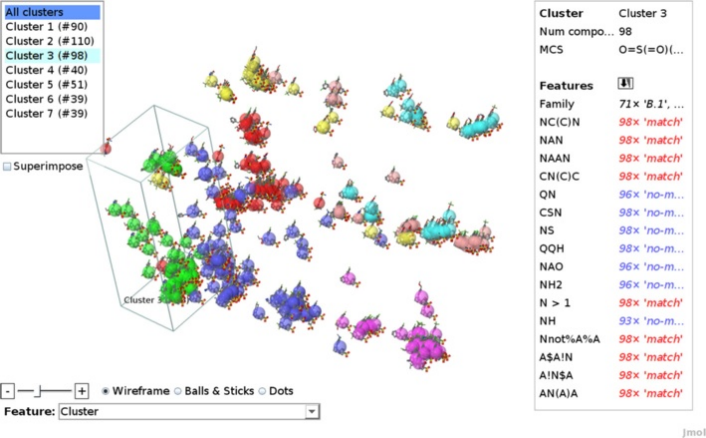
**The COX-2 dataset is clustered into 7 clusters.** The compounds are highlighted according to their cluster assignment. Cluster 3 is selected (as indicated by the box), and the a summary of feature values is shown on the right-hand side. Spheres are employed for highlighting (instead of changing the color of the structure).

The information panel on the right-hand side of the screen has been extended and shows the properties of the currently selected cluster or compound, or of the entire dataset. In particular, all features in the dataset and the (mean) feature value of the currently selected element are presented. When features are selected for highlighting, the compounds are colored according to their feature value (Figure 4). A novelty is that the compound list (at the left-hand side of the viewer in Figure 4), is completed with feature values for each compound and sorted according to this value. This extension (referred to as (*a*) in Table 1) facilitates the identification of compounds with the highest or lowest features values. Instead of changing the color of the whole compound, feature values can now be highlighted using translucent spheres that are overlayed over each compound (see e.g. Figure 5). This preserves the standard atom coloring of compounds. Moreover, we added the option to highlight two features at once by adding a second, flattened spheroid (Figure 1). This novel functionality (see Table 1 (*b*)) can be used to directly compare the values of two features.

#### 3.1.1 Measuring embedding quality and embedding stress

It is not always possible to compress the feature space without loss of information. This is especially the case if many diverse and/or uncorrelated features are selected by the user. CheS-Mapper 2.0 computes a global embedding quality measure that describes how well the feature values are reflected by the 3D positions of the compounds (see Table 1 (*c*)). A standard stress function [15] has the disadvantage that it cannot be used to compare the embedding of different datasets: it is commonly defined as the sum of squares between the pairwise distances in the high-dimensional representation (feature values) and the low-dimensional representation (3D positions). Instead, CheS-Mapper computes the Pearson’s product-moment correlation coefficient between the distance pairs. The distances based on the feature values are computed using the (dis-)similarity measure of the selected embedding algorithm. The 3D distance values are computed using the Euclidean distance, resembling the human user’s perception of distances between compounds in 3D space. The embedding quality ranges from 1 (perfect correlation) to 0 (no correlation) to -1 (negative correlation). A warning is given to the user if the correlation is below 0.6, corresponding to moderate or weak embedding quality [36].

In some use cases, the overall embedding is good, apart from some outlier compounds that might have largely differing feature values. Therefore, CheS-Mapper provides the embedding stress for each compound. We define embedding stress as 1− Pearson’s correlation coefficient between the distance pairs of the corresponding compound to all other compounds in the dataset. Accordingly, compounds with a value close to 0 have low embedding stress, whereas a value close to 1 corresponds to high stress.

The global embedding quality is presented to the user at the top right-hand side of the viewer (see Figure 2). The embedding stress can be highlighted with the drop down menu coloring compounds with low embedding stress in blue, while compounds with high embedding stress are colored in red.

CheS-Mapper can also compute and highlight the distance from all compounds in the dataset to a particular compound, based on the features and distance measure that were used for the 3D embedding. When a good 3D embedding is feasible, this distance mirrors the proximity between compounds in 3D space. However, if the 3D embedding is poor, or a particular compound has a high embedding stress, this function allows to determine the nearest neighbors for a particular compound.

#### 3.1.2 Determination of common properties of compounds

When exploring a clustered dataset with CheS-Mapper, a common task is to identify the reasoning of why compounds are assigned to the same cluster. Similarly, the user might want to determine why two particular compounds are located close to each other in 3D space. In both cases, the user is looking for common properties of groups of compounds that separate these instances from the remainder of the dataset.

**Figure 6:**
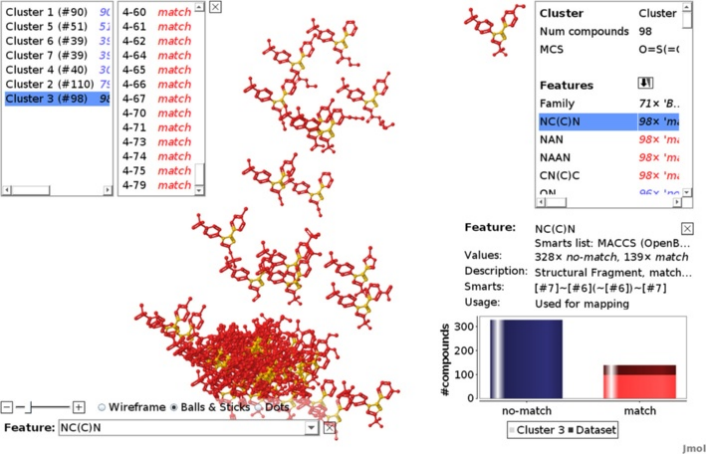
**Cluster 3 of the COX-2 dataset is selected.** Only compounds of the selected cluster 3 are visible. At the top left-hand side, the cluster and compound list can be used to select another cluster or a compound within the active cluster. The feature list of cluster 3 at the top right-hand side is sorted according to specificity. The structural feature *NC(C)N* is very specific, as it matches mostly compounds within this cluster (as indicated by the bar chart). The fragment is highlighted in each compound structure with orange color.

This kind of information is dynamically provided by CheS-Mapper 2.0 (see Table 1 (*d*)), even for large datasets with numerous features: the feature list (on the right-hand side of the viewer) is sorted depending on the currently selected cluster or the currently selected compound/s. In more detail, the list is sorted in descending order according to the specificity of the feature values of the selected elements. Hence, the *most important* features that distinguish the selected compounds from the remaining dataset can be found at the top of the list. Examples are given in Figures 3 and 6. The specificity of features is computed by comparing the feature values of the selected elements to the feature values of the entire dataset. To this end, statistical tests are applied and the features are sorted in ascending order according to the *p*-value: low *p*-values indicate that the tested distributions differ from each other and high *p*-values indicate similar distributions. A *χ*^2^-test is exercised for nominal feature values [37]. For numeric features, we employ one-way analysis of variance (ANOVA) [37]. When comparing the numeric feature value of a single compound to the overall feature values distribution, the ANOVA test is not applicable. We do therefore apply the *χ*^2^-test on binned numerical data to compute the *p*-value for numeric features of single compounds^a^.

#### 3.1.3 Analysis of activity space and activity cliffs

**Figure 7:**
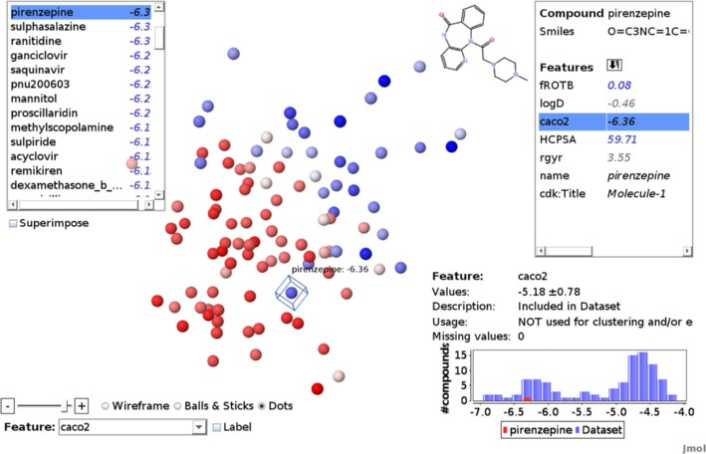
**Highlighting the endpoint of the Caco-2 permeability dataset.** Selecting the endpoint feature shows the activity space (or landscape). The endpoint was not employed for 3D embedding. The depiction setting is set to the new depiction option *Dots*. The compound *pirenzepine* is selected (indicated with a blue box), the compound information including a 2D image of the compound is shown on the right-hand side. The compound forms an activity cliff, as its endpoint value differs from its neighbor compounds.

CheS-Mapper can be employed for various purposes, including the analysis of datasets without endpoint activity information. However, the typical use cases are the analysis of (Q)SAR information and of the activity landscape of a small molecule dataset. Commonly, activity landscapes depict activity values in *2* feature dimensions. As CheS-Mapper provides an additional third dimension (3D space), the term activity space is more appropriate. Inspecting the activity space of a dataset with CheS-Mapper requires that the endpoint values are stored in the dataset, but not employed for 3D embedding. Highlighting the endpoint feature in the CheS-Mapper viewer presents the activity space, as shown in Figure 7. The user can detect activity cliffs by locating compounds that stand out in the color coding, when compared to neighboring compounds in nearby 3D space. This indicates that these compounds have differing endpoint values, yet similar feature values.

Additionally, CheS-Mapper provides a new functionality to automatically reveal activity cliffs by computing the Structure-Activity Landscape Index (SALI) (see Table 1 (*e*)). The SALI value is computed for pairs of compounds and a high SALI value indicates that a pair resembles an activity cliff [21]. We transform the SALI value matrix to a feature (with a single value for each compound) by calculating the mean SALI values for each compound. Additionally, CheS-Mapper provides the standard deviation and maximum pairwise SALI values. Hence, compounds forming activity cliffs can be determined and inspected. Activity cliffs can further be studied by investigating common properties of a particular compound and its neighboring compounds. For instance, the user might detect that the features that have been selected for embedding cause an activity cliff, as some active and inactive compounds cannot be distinguished [38].

#### 3.1.4 CheS-Mapper extension for KNIME

**Figure 8:**
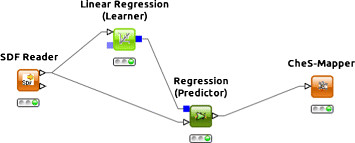
**A simple KNIME workflow including the CheS-Mapper visualization node.** CheS-Mapper is employed to visually inspect the modeled activity of linear regression, based on properties that are stored in a SD-File.

We have included CheS-Mapper into KNIME (Konstanz Information Miner), a graphical framework for data analysis [39] (see Table 1 (*f*)). The framework has various extensions for cheminformatics and machine learning, and can therefore be employed for (Q)SAR modeling. The CheS-Mapper node for KNIME is a pure visualization node that envisions data which has been arbitrarily processed within KNIME (see http://tech.knime.org/book/ches-mapper-node-for-knime-trusted-extension). A simple example for using CheS-Mapper within KNIME is shown in Figure 8. In this case, CheS-Mapper is applied for analyzing prediction results of a regression model (follow the link above to find a detailed description of this workflow).

#### 3.1.5 Store configuration for chemical space mapping

A novel functionality in CheS-Mapper is that the current mapping settings, which are configured in the wizard, can be stored to a file and shared with co-workers. This is especially helpful when exploring the same dataset in a team, as it preserves different useful configurations like e.g. the selected features, clustering, and 3D embedding settings. The configuration includes the random seed for randomized approaches (like e.g., *k*-means clustering), to ensure that the mapping settings always produce the same mapping result.

### 3.2 Visually validating (Q)SAR models in CheS-Mapper 2.0

Visual validation describes the graphical inspection of (Q)SAR model validation. Initially, a (Q)SAR model is built and validated on a compound dataset. The dataset is then visualized with CheS-Mapper, using the same features for embedding that were used to validate the (Q)SAR model. Additionally, the endpoint values and the prediction results of the model are employed within the visualization. Consequently, CheS-Mapper allows the inspection of actual and predicted activity in the feature space. The visual validation approach can be re-iterated to take possible insights from the visualization steps into account for model re-building. However, re-iteration should be handled with care to prevent model overfitting or chance correlation (as discussed below).

In general, the proposed method can be performed with an arbitrary validation scheme, like e.g. test set validation or repeated *k*-fold cross-validation. The predicted endpoint can be qualitative or quantitative (i.e. classification and regression models can both be analyzed).

#### 3.2.1 Mapping the feature space of the (Q)SAR model

When performing visual validation with CheS-Mapper the dataset is mapped into 3D space based on the same features as employed by the (Q)SAR modeling. Hence, when exploring the embedded dataset with CheS-Mapper, the user is provided an intuitive view on the feature space that is employed as input for the (Q)SAR approach. Depending on the model, it is likely that similar compounds are predicted with similar activity values. For example, when performing classification, compounds will be assigned the same class value if they are on the same side of the decision boundary.

#### 3.2.2 Comparing actual and predicted activity

The main idea of visual validation is that the user is able to simultaneously compare the experimental and predicted endpoint values in the feature space. These values can be highlighted in CheS-Mapper, by coloring the compounds according to their actual or predicted activity value. Moreover, the application can highlight both activity values at the same time (see Figure 1). Hence, the user is able to identify compounds that have been misclassified by the (Q)SAR model and investigate possible reasons. A model might under-fit the target concept which results in an overly smooth predicted activity space, or if classification is applied, in large areas of compounds with same predicted class. In contrast, the (Q)SAR model might be too complex and overfits the data. Furthermore, the model could fail in predicting compounds that form activity cliffs. As described above, activity cliffs can be detected and investigated with CheS-Mapper.

Directly highlighting the prediction error (instead of individually selecting predicted and actual endpoint values) allows the selection of groups of misclassified compounds with CheS-Mapper. Therefore, the user can detect common properties of these compounds and investigate possible weaknesses of a model. When performing classification, the probability (or confidence) of a classifier for each prediction is a useful extension for the visualization of decision boundaries. These are areas in feature space where the compound predictions change from one class to another (e.g. from active to inactive). As some (Q)SAR models do not provide probability estimates, repeated validation can overcome this limitation, as described in the next section.

#### 3.2.3 Visually validating repetitive validation approaches

Arbitrary (Q)SAR modeling software can be employed for visual validation. Modeling and validation results have to be stored in the CheS-Mapper input dataset file (e.g. SD-File or CSV-File), in addition to the model input features and actual endpoint values. In more detail, the following validation results should be available for each predicted compound:

 Predicted endpoint value (class or numeric regression value)

 The prediction error (difference between actual and predicted endpoint value, optionally the squared-error for regression)

 A probability or confidence measure (if available)

 The applicability domain value (inside/outside, if available)

As mentioned above, our presented visual validation approach can be employed using any arbitrary validation technique. Depending on the selected validation approach, compounds can even be predicted multiple times. When applying a single training test set split, test set compounds will be predicted only once. A *k*-fold cross-validation yields a prediction for every compound in the dataset. When using a repetitive sampling scheme, like bootstrapping or a *n*-times repeated *k*-fold cross-validation, compounds are predicted multiple times by (Q)SAR models trained on different subsets of the data. As mentioned in our previous work [40], a repetitive validation approach should be preferred for small datasets to avoid overfitting (caused by e.g., “parameter fiddling”). In particular, visual validation should not be used to maximize the prediction accuracy on a single test set, as this would most likely not improve the predictivity of the (Q)SAR model. For visual validation, each repetition (or run of the validation approach) could be inspected separately, but it is more reliable to inspect the aggregated result. Consequently, multiple predictions for each compound should be combined as follows:

 For numeric predictions, the mean predicted value is preferable. When performing binary classification, the prediction can be transformed to continuous values between 0 and 1 (this is e.g. the ratio how often a class was predicted as active). For classification with multiple classes, the majority class prediction or a *inconclusive value* could be used.

 The prediction error can be averaged with standard techniques, like accuracy for classification and root-mean-squared-error for regression.

 The mean of the probability or confidence is adequate.

 The applicability domain value should be transformed to a continuous 0-1 value, corresponding to the ratio of how often the compound was inside the applicability domain.

#### 3.2.4 Limitations

As already stated before, the exact reasoning behind predictions is hard to comprehend for humans in many (Q)SAR modeling approaches. A prediction algorithm whose predictions can be easily understood with CheS-Mapper is a *k*-Nearest Neighbor algorithm, as illustrated in Figure 1. Nevertheless, even if predictions are not comprehensible to researchers, inspecting and comparing actual and predicted activity values can provide valuable information (as discussed above).

Another limitation of our approach is that it relies on a good mapping of the data into 3D space. Often, CheS-Mapper can achieve high embedding quality as it employs a third dimension (compared to standard 2D mapping approaches) and provides various embedding algorithms with configurable distance measures. However, dimensionality reduction without loss of information is not always feasible, especially on large and diverse datasets and when applying non-redundant and uncorrelated descriptors (which is preferable for (Q)SAR modeling). In these cases, CheS-Mapper yields an oversimplified and compressed view of the feature space and the spatial distance does not resemble the descriptor-based similarity for all compounds. As described above, CheS-Mapper allows detecting poorly embedded compounds by computing and highlighting embedding stress. Moreover, the descriptor-based distance to a dedicated compound can be calculated to detect neighboring compounds. An additional functionality that helps to overcome this limitation is the computation of activity cliffs, which is not dependent on the embedding.

## 4 Results

We employ visual validation with CheS-Mapper to analyze real world datasets that include experimentally derived activity endpoints. Table 1 provides an overview of the subsequent use cases. We show how researchers can employ the new functionalities to investigate the correlation between feature values and activity values. Furthermore, we explore (Q)SAR model prediction and validation results with CheS-Mapper, and inspect different applicability domain approaches that exclude different compounds from a dataset. Please note that all datasets and configuration settings are provided in Additional file 1. Moreover, an additional use case is described in Additional file 2 on visually validating (Q)SAR modeling of the same endpoint with different sets of descriptors.

### 4.1 Compare (Q)SAR models for Caco-2 permeation

In our earlier work [13], we applied CheS-Mapper to visualize and verify work on the correlation of Caco-2 permeation with simple molecular properties [41]. We have visually verified this correlation by using the four molecular properties for 3D embedding of the dataset that includes 100 structurally diverse compounds. We repeat this experiment to demonstrate CheS-Mapper 2.0 functionalities, and subsequently compare two (Q)SAR modeling approaches applied to this data.

**Figure 9:**
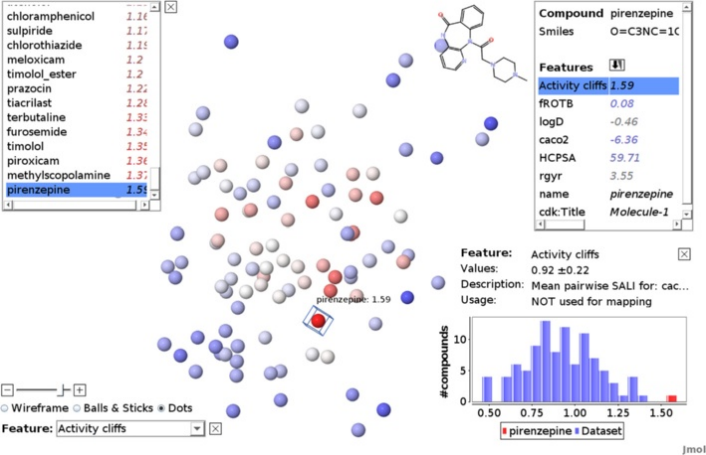
**Highlighting activity cliffs within the Caco-2 permeability dataset.** The mean SALI values are computed and highlighted. The compound *pirenzepine* is selected. It is the compound with the highest mean SALI value, as indicated in the histogram (at the bottom right-hand side) and in the compound list on the left-hand side (*pirenzepine* is at the bottom of the sorted compound list). Alternatively, the feature with the maximum SALI value or the standard deviation can be selected: *pirenzepine* has the highest maximum SALI value in the dataset (4.01) and the second highest standard deviation (0.8).

The result of the embedding can seen in Figure 2. When highlighting *logD*, we observe that compounds with similar *logD* values are close to each other, as this feature was used for embedding (see Figure 4). In fact, the dataset is almost perfectly embedded into 3D space using principal components analysis (*Pearson* :0.99). This is due to the fact that the number of dimensions has to be reduced only by one: from 4 molecular descriptors to a 3 dimensional space. Additionally, inter-correlation of feature values simplifies the dimensionality reduction (e.g. most compounds with high feature values of *high charged polar surface area (HCPSA)* have a low *logD* value). Even though the endpoint was not used for embedding, compounds that are close to each other tend to have a similar endpoint value as exemplified in Figure 7, i.e. the activity space (or landscape) is smooth. This supports the findings of Hou et al. [41], the endpoint is indeed correlated to the feature values presented in the dataset. In our previous assessment, we were also able to visually detect the compound *pirenzepine* (selected in Figure 7; the viewer has zoomed in on this compound in Figure 3). Being part of the training dataset in the original article, *pirenzepine* is the compound with the highest training error. It attracted our attention in CheS-Mapper, as it has a relatively low endpoint value (and is therefore colored in blue), but is spatially close to compounds with high endpoint values (colored in red). Hence, it is part of an activity cliff as its endpoint value differs from compounds with similar feature values. A new function of CheS-Mapper is to automatically locate activity cliffs. Therefore, compounds can be sorted and highlighted according to their pairwise SALI values. For this dataset, *pirenzepine* stands out as the compound with the highest mean and second highest standard deviation (see Figure 9).

We use two different (Q)SAR approaches to model Caco-2 permeability. Instead of adopting the training test split that was used in the original article [41], we apply a leave-one-out cross-validation procedure to compare support vector regression and simple linear regression. The visual validation workflow is implemented with the CheS-Mapper extension for KNIME [39] and is described in Additional file 3. The visual validation with CheS-Mapper shows, as expected, that *pirenzepine* has the highest prediction error in simple linear regression and the second highest prediction error in support vector regression. According to statistical validation with KNIME, the *R*^2^ value of support vector regression is 0.54, simple linear regression attains a value of 0.51.

**Figure 10:**
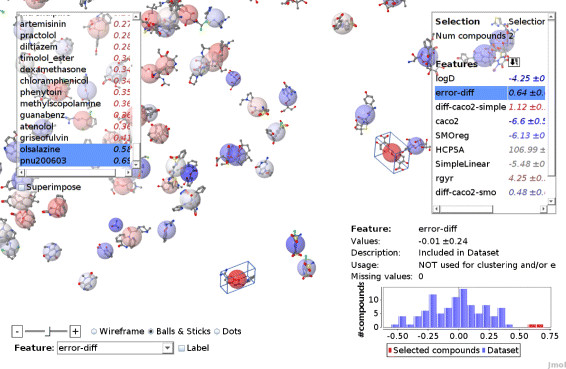
**Inspecting the prediction error difference of two (Q)SAR approaches.** The prediction error difference (*error*_*simple*-*linear*_− *error*_*support*-*vector*_) of two regression approaches for the Caco-2 dataset is selected. Simple linear regression performed especially bad for the two selected compounds (*olsalazine* and *pnu*200603). Both compounds have a very low *logD* value (*logD* is the top feature in the feature list on the right-hand side).

We investigate the reason for the predictivity difference with CheS-Mapper. We highlight the prediction error difference for each compound to determine which compounds are predicted more accurate by which approach (see Figure 10). The distribution of the prediction error difference is depicted as histogram in the figure: it indicates that the overall less accurate result of simple linear regression is mainly due to the prediction of two compounds. Using CheS-Mapper, we can easily determine common properties of these two compounds (*pnu200603* and *olsalazine*): the two compounds have the lowest *logD* feature values in the dataset. Consulting the original publication confirms the assumption that the *logD* value causes the high prediction error in linear regression. *logD* is treated differently from the other three input features, as the function to predict the endpoint value includes a cutoff for high and low *logD* values^b^. This cannot be modeled by simple linear regression, and causes the inferior predictivity compared to support vector regression.

**Figure 11:**
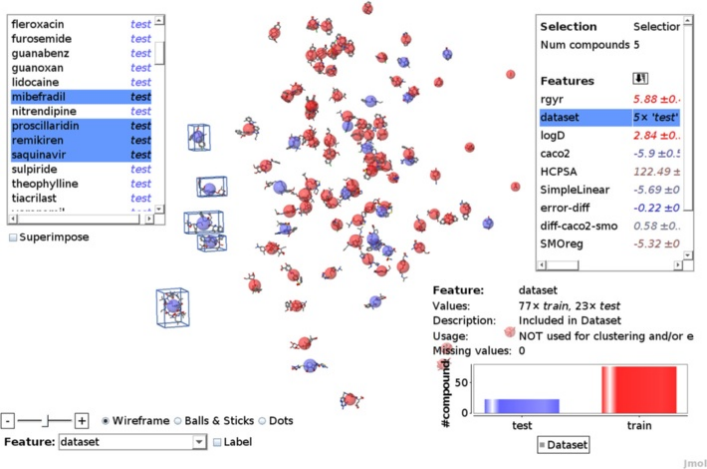
**Visualizing the selected test set compounds of the Caco-2 dataset.** The screen-shot shows the distribution of training and test compounds in the feature space. The 5 selected test compounds share in particular high values for *radius of gyration* (*rgyr*) and *logD*. As a result, both features are at the top of the feature list on the right-hand side.

Finally, this use case demonstrates shortcomings of external test-set validation compared to cross-validation. We have previously shown [40] that not using the complete data for building the final (Q)SAR model, which is used to predict unseen compounds, will yield a less predictive model. This is especially the case if all compounds of an entire region of the data set are removed from the model building set and split away into the test set. There is no information given on how the test set split was performed in the original article, however, the test set includes 5 neighboring compounds (see Figure 11). Accordingly, each of the 5 compounds is predicted with a higher error by a support vector model build on the training data (mean error 0.86) compared to the leave-one-out approach (mean error 0.58). This indicates that the final model of external test-set validation has a lower predictivity for similar unseen compounds and/or has a smaller applicability domain.

### 4.2 Structural clustering of COX-2 Data

We apply CheS-Mapper to structurally cluster and embed a dataset using MACCS keys [42], and investigate if these structural fragments are suitable to model the inhibitory potential of the dataset compounds. The dataset contains 467 COX-2 inhibitors [43],[44], that have been tested for the selective inhibition of the human enzyme Cyclooxygenase-2 (COX-2). The experimentally derived activity of each compound is stored in the dataset as *IC*_50_ value (half maximal inhibitory concentration). The inhibitors are structurally very similar, as they have to fit the active site of the COX-2 enzyme. We apply visual validation using MACCS keys, as a recent attempt to model the dataset with these features failed [45]. As proposed in previous work [43],[44], we transform the numeric endpoint to a binary nominal endpoint. Equal-frequency discretization yields 234 active compounds with *IC*_50_≤0.12*μMol*. A random forest classifier based on the structural fragments achieves 0.75 accuracy, validated with a 10-times repeated 10-fold cross-validation. To compute the structural fragments, CheS-Mapper matches the 166 SMARTS fragments of the MACCS list with the dataset compounds. This generates 97 nominal features with a minimum frequency of 10.

**Figure 12:**
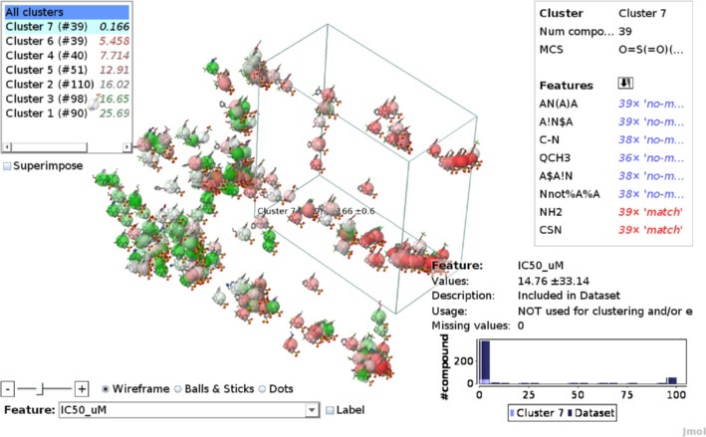
**Highlighting*IC***_**50**_** values within the COX-2 dataset.** The endpoint value of the COX-2 dataset is selected, showing the activity space (or landscape). A new function of CheS-Mapper has been used to modify the highlighting colors, using red for active compounds with low feature values and applying a log-transformation. Compounds with high feature values are located predominantly on the right-hand side. The selected cluster 7 includes many active compounds.

For visual validation, we employ the features as input for hierarchical clustering using the dynamic tree cut method that is included in CheS-Mapper to automatically compute the number of clusters [46]. Sammon’s non-linear mapping is used for 3D-embedding [47]. We employ the Tanimoto similarity measure for the clustering and embedding techniques. Finally, we enable 3D alignment according to the maximum common subgraph (MCS). The mapping result is shown in Figure 5 and divides the dataset into 7 clusters of different sizes (ranging from 39 to 110 compounds). Moreover, CheS-Mapper gives a warning to the user that 467 compounds have been mapped to only 333 distinct positions in 3D space due to identical feature values of numerous compounds. Hence, several of the structurally similar compounds cannot be distinguished with the 97 fragments matched by the SMARTS list (as discussed in detail below). Even though the dimensionality reduction cannot be achieved without loss of information, the distance in the 3D-space resembles the Tanimoto distance well for most compound pairs (*Pearson*: 0.89). Highlighting the target endpoint (see Figure 12) shows that clustering and embedding apparently separate active and inactive compounds. Compounds with low *IC*_50_ values are mostly on the right-hand side (drawn in red), and compounds with high values (green) mostly on the left-hand side. Similarly, the endpoint values of the clusters do largely differ from each other: as an example, 34 of 39 compounds in cluster 7 are categorized as active. Accordingly, cluster 7 has a much lower mean *IC*_50_ value compared to other clusters (see cluster list on the left-hand side of Figure 12).

When investigating the clustering result, the user is usually interested in the most specific features that *define* a cluster. As the features are sorted according to specificity, CheS-Mapper makes this information easily accessible. As an example, the most specific structural feature for cluster 3, that comprises 98 predominantly inactive compounds, is the SMARTS fragment *NC(C)N*. It matches each compound of this cluster. In contrast, most of compounds in the dataset (328 of 467) do not contain this fragment. This can be seen in (the chart of) Figure 6, where the view has zoomed in on cluster 3, and the corresponding feature was selected.

**Figure 13:**
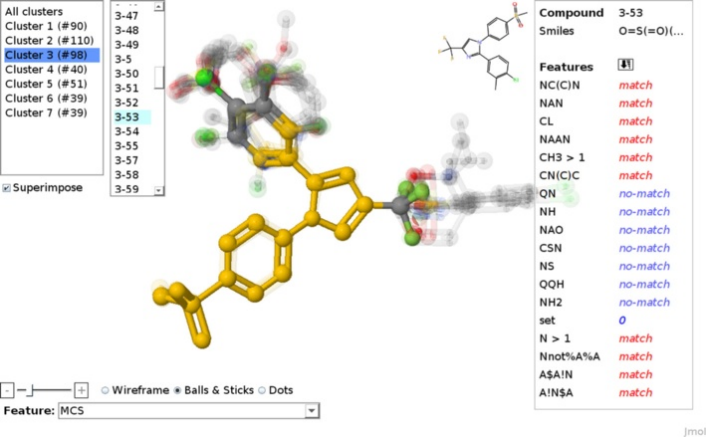
**Superimposition of compounds that are aligned in 3D space.** Cluster 3 of the COX-2 dataset has been aligned in 3D according to the *maximum common subgraph* (MCS). In this screen-shot, the compounds are superimposed to compare the compound structures. The MCS feature is selected and therefore highlighted in orange. The depiction setting for compounds is *Balls & Sticks*.

Furthermore, we applied 3D alignment using the maximum common subgraph. As this dataset consists of structurally very similar compounds, large common fragments have been found. Cluster 3 shares the fragment *O=S(=O)(c1ccc(cc1)n2ccnc2(cc))C*, that is highlighted orange in Figure 13. The superimposition simplifies the structural comparison of clusters within the dataset.

When computing activity cliffs for this dataset, CheS-Mapper reveals that 95 compounds share equal feature values with another compound in the dataset that has the opposite nominal endpoint value. Consequently, many of these compounds are misclassified by the (Q)SAR approach. For instance, these compounds account for the majority of compounds that are misclassified in every single repetition of the cross-validation (34 of 51 compounds). We conclude that the fragments based on the MACCS keys do provide valuable (Q)SAR information, but cannot distinguish numerous active and inactive compounds. This probably caused the bad modeling performance in the work cited above [45]. Including additional fragments could aid to improve the (Q)SAR model.

### 4.3 Applicability domains for fish toxicity prediction

As final visual validation use case, we examine different applicability domain (AD) approaches. The use of ADs is a necessity due to the vast size of chemical space and to assure that a (Q)SAR model only *interpolates* but does not *extrapolate*. There exist various AD methods [48] that exclude different compounds from prediction. AD models can be regarded as prediction algorithms, statistical models that predict whether a compound is inside, or outside of the model domain. Similar to statistical (Q)SAR models, single predictions may be hard to reproduce.

**Figure 14:**
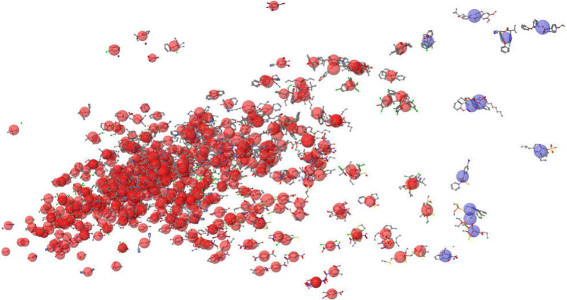
**Applicability domain (AD) computed with a centroid distance-based method.** A centroid distance-based method using Euclidean distance is applied to the Fathead Minnow Acute Toxicity, based on five physico-chemical descriptors. Compounds that are inside the AD are highlighted in red, compounds that are outside the AD are colored blue.

**Figure 15:**
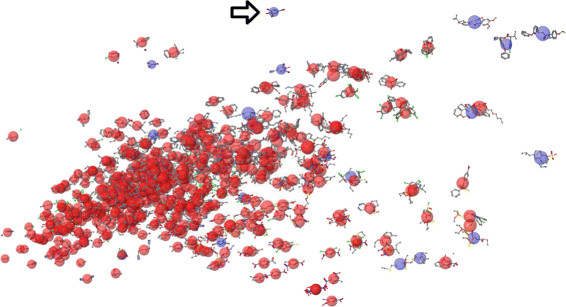
**Applicability domain (AD) computed with Leverage method.** The Leverage method is applied to the Fathead Minnow Acute Toxicity, based on five physico-chemical descriptors. Compounds that are inside the AD are highlighted in red, compounds that are outside the AD are colored blue. The excluded compounds *2,4,6-Triiodophenol* is marked.

**Figure 16:**
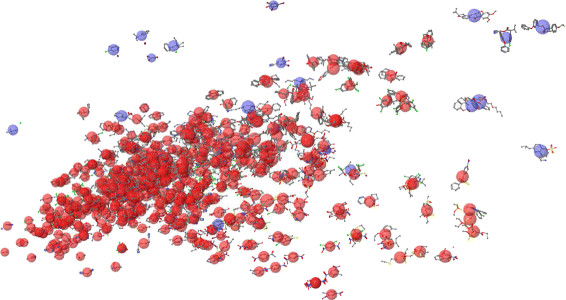
**Applicability domain (AD) computed with a*k*-Nearest-Neighbor distance-based method.** The *k*-Nearest-Neighbor distance-based method using Euclidean distance is applied to the Fathead Minnow Acute Toxicity, based on five physico-chemical descriptors. Compounds that are inside the AD are highlighted in red, compounds that are outside the AD are colored blue.

As example we select a fish toxicity dataset (Fathead Minnow Acute Toxicity) [49], published by the US Environmental Protection Agency (EPA). The endpoint is highly correlated to physico-chemical (PC) descriptors. We have used five physico-chemical descriptors as a basis for the AD computation: molecular weight, number of bonds, octanol/water partition coefficient (logP), topological polar surface area (TPSA), and molar refractivity. Figures 14, 15 and 16 show three different AD methods applied to this dataset. The compound embedding is the same for all methods, and was performed with Sammon’s mapping using default settings. The embedding quality is excellent (*Pearson*: 1). CheS-Mapper reveals that the PC feature values are correlated in this dataset, especially the values of molecular weight, number of bonds and molar refractivity (compounds on the left-hand side of the figures have low values, compounds on the right-hand side have high feature values).

Without going into detail regarding the functionality of the AD methods, we describe some characteristics and (dis-)advantages of the AD approaches that can be investigated using CheS-Mapper. The distance-based approach using the Euclidean distance to the centroid is shown in Figure 14: if compounds differ too much from the *centroid compound* (a virtual compound with mean feature values), they are excluded from the AD. As for most AD methods, the user has to set the threshold manually (we have selected 3 times the mean distance to the centroid). One disadvantage of this approach is that outliers with extreme values for a single feature are often not excluded when features are correlated. This is circumvented by the leverage approach [50]. This is a centroid distance based approach as well, but neglects inter-correlation of feature values (by computing the distance using the diagonal elements of the hat matrix). As a result, the marked compound at the top center of the embedding (*2,4,6-Triiodophenol*) is excluded from the AD with the leverage approach (see Figure 15), but not with the Euclidean distance centroid approach. It is the second heaviest compound in the dataset and therefore an outlier, but it has moderate number of bonds and moderate molar refractivity. Both centroid distance based approaches have the disadvantage that in diverse datasets not only individual separate outliers are removed, but also groups of outlying, similar compounds (see bottom right-hand area in the figures). The *k*-nearest neighbor distance based AD approach (Figure 16) overcomes this disadvantage: compounds are only excluded from the AD if the distance to *k* nearest neighbors is too high (we set *k* to 3, and the mean distance has to be ≤ 3 times the mean *k-NN* distance).

Which of the three approaches is more suitable for this dataset depends on the applied (Q)SAR model. Therefore, CheS-Mapper helps to understand AD methods and allows inspecting compounds that are excluded from the dataset.

## 5 Discussions and conclusions

In this work, we presented how visual validation can be performed with CheS-Mapper 2.0, an improved and updated version of our 3D viewer for small molecule datasets. In particular, CheS-Mapper now allows to analyze activity cliffs, to detect common properties of subgroups of compounds within the dataset, and to calculate the 3D embedding quality.

In our work, visual validation is understood as the graphical analysis of (Q)SAR model validation results. Therefore, the predicted dataset is embedded into 3D space, based on the same features that have been employed for (Q)SAR modeling. The highlighting functionality of CheS-Mapper allows to compare the predictions to the actual activity values within the feature space. The user can in particular inspect how the model predicted compounds that form activity cliffs. Visual validation can aid the (Q)SAR model developer to select appropriate features, to detect possible inconsistencies within the data, and to investigate strengths and weaknesses of the employed (Q)SAR approach. Re-iterating (Q)SAR modeling, statistical validation and visualization can improve the model predictivity and supports the researcher in mechanistically interpreting model predictions, which is an important requirement for the acceptance of (Q)SAR models as alternative testingmethod.

In the future, we consider adding model building functionalities to CheS-Mapper, in order to build and visually validate (Q)SAR models directly within the software. Future work might also include applicability domain calculation, *jittering* of multiple compounds that are mapped to the same 3D position, and the implementation of an additional java-based dimensionality reduction technique.

## 6 Availability and requirements

***Project name:*** CheS-Mapper ***Project home page:***http://ches-mapper.org***Operating system(s):*** Cross-platform ***Programming language:*** Java with Java Web Start support (can be started from a web browser) ***Other requirements (optional):*** For extended functions OpenBabel [34] and R [35] (both free). ***License:*** GNU GPL v3 ***Any restrictions to use by non-academics:*** No additional. For proper use, guidance and maintenance, contact ches-mapper@informatik.uni-freiburg.de

## 7 Endnotes

^a^ The Apache Commons Mathematics Library is employed for statistical testing (http://commons.apache.org/math). To test the specificity of numeric features of single compounds, equal-width binning is applied with initially 20 bins. Hence, the numeric data is divided into categories using 20 intervals of equal width. If intervals without any compounds exist, the number of intervals is decreased by one and the binning method is reapplied. To produce a compact data representation, this process is iteratively repeated until no empty bins exist.

^b^ The formula from [41] to predict the endpoint is *logP*_*eff*_=−4.358+0.317×*min*(*max*(−1.8,*logD*),2.0)−0.00558×*HCPSA*−0.179×*rgyr*+1.074×*f*_*rotb*_.

## Abbreviations

(Q)SAR: (Quantitative) structure-activity relationship

CheS-Mapper: Chemical Space Mapper

CDK: Chemistry Development Kit

SMARTS: Smiles arbitrary target specification

SALI: Structure-activity landscape index

PC: Physico-chemical

AD: Applicability domain

## Competing interests

The authors declare that they have no competing interests.

## Authors’ contributions

MG implemented and designed the CheS-Mapper software. AK and SK provided the idea for CheS-Mapper and its application to (Q)SAR validation, as well as valuable guidance throughout the implementation. All authors read and approved the final manuscript.

## Additional files

## Supplementary Material

Additional file 1 **Datasets and CheS-Mapper configuration.** A zip-file containing the data used in this article for the use-cases and screen-shots. Click here for file

Additional file 2 **Investigate input features for carcinogenicity models.** A description of the KNIME workflow that is used to visually compare a leave-one-out cross-validation of the Caco-2 data. Click here for file

Additional file 3 **A KNIME Workflow for visually validating LOO-CV.** A description of the KNIME workflow that is used to visually compare a leave-one-out cross-validation of the Caco-2 data. Click here for file
