# Supplementary material for: CheS-Mapper 2.0 for visual validation of (Q)SAR models
Source: J Cheminform. 2014 Sep 23;6:41. doi: 10.1186/s13321-014-0041-7 (PMC4186979; doi:10.1186/s13321-014-0041-7)
Supplement: Additional file 2 — Investigate input features for carcinogenicity models. A description of the KNIME workflow that is used to visually compare a leave-one-out cross-validation of the Caco-2 data. [file s13321-014-0041-7-S2.pdf]

SOFTWARE

Additional File 2 — Use Case: Investigate Input Features for Carcinogenicity Models

Martin Gütlein[1], Andreas Karwath[2] and Stefan Kramer[2*]

*Correspondence: kramer@informatik.uni-mainz.de
[2] Information Systems, Institut für Informatik, Johannes Gutenberg - Universität Mainz, Staudingerweg 9, D-55128, Mainz, Germany
Full list of author information is available at the end of the article

Additional file for the article "CheS-Mapper 2.0 for Visual Validation of (Q)SAR models"

We visually validate the effect of exchanging the descriptors used by a (Q)SAR algorithm. To this end, we select a subset of the Carcinogenic Potency Database (CPDB) [1] for various species. The database contains 86 compounds that have an activity value for hamster carcinogenicity assigned (active or inactive). We compute two different sets of features for these compounds with CheS-Mapper: 308 physico-chemical (PC) descriptors using CDK and Open Babel, and 287 structural fragments. The structural features have been calculated by matching the compounds with three predefined SMARTS lists included in Open Babel. We choose the random forest implementation from the WEKA workbench as classification algorithm and compare two different approaches: *(Q)SAR-1* is built using only the physico-chemical descriptors, while *(Q)SAR-2* exploits a combination of both feature sets. We apply a 5-times repeated 10-fold cross-validation to validate both variants. *(Q)SAR-2* achieved a classification accuracy of 0.75, and significantly outperformed *(Q)SAR-1* that had a classification accuracy of only 0.67. Apparently, using both feature types allows to build a more predictive model.

As CheS-Mapper's 3D embedding is based on the features, we start the program twice to (simultaneously) compare the effect of using different feature sets. When highlighting the actual endpoint value, we note that the compounds are roughly separated according to their class value. The separation (and thus the decision boundary) is less distinctive when using only PC features (Figure 1) compared to adding structural fragments (Figure 2). This indicates that it is easier for *(Q)SAR-2* to predict the endpoint, than for the *(Q)SAR-1* approach. Comparing the misclassifications of both approaches, we detect two compounds that have always been correctly classified by *(Q)SAR-2*, but not by *(Q)SAR-1*.

The inactive compound *Isonicotinic acid* (DSSTox-RID 20757) is selected in Figure 2 (marked with a label and drawn as 2D picture at the top right-hand side). In the embedding based on both feature types it is located in entirely inactive space. It is correctly classified by *(Q)SAR-2* in 5 of 5 repetitions of the cross-validation. In contrast, this compound was misclassified as active 2 out of 5 times by *(Q)SAR-1*. As previously described, the feature list at the top right-hand side is sorted according to specificity. Hence, *carboxylic acid* is the structural feature that distinguishes this compound the most from the remaining dataset compounds. With the help of CheS-Mapper, we detect that this compound is one of 6 compounds in the dataset

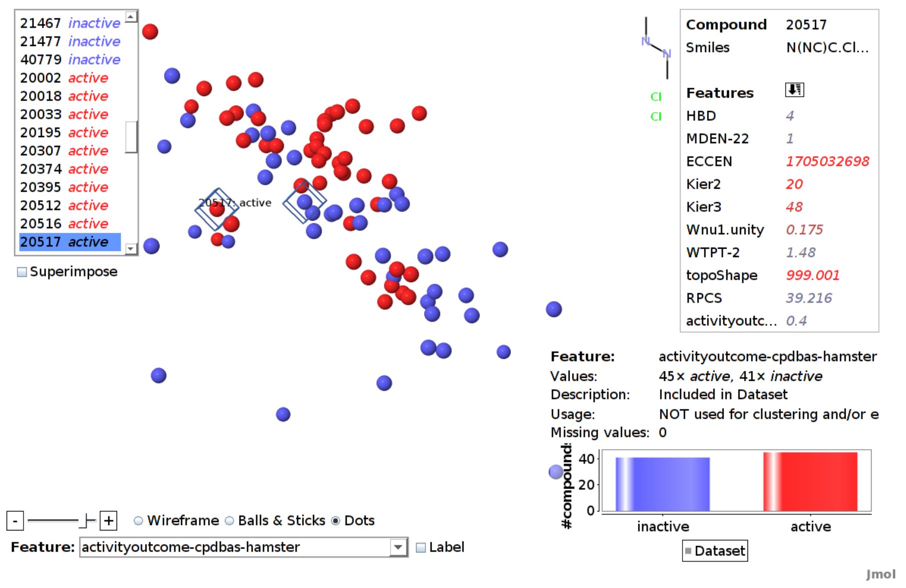

Figure 1 Applying PC descriptors to embed the CPDB hamster dataset.
Compound 20517 (*active*) is selected, compound 20757 (*inactive*) is also marked with bounding boxes. The actual activity values are highlighted. (Dots are drawn instead of compound structures to illustrate the distribution of class values and the selection of compounds.)

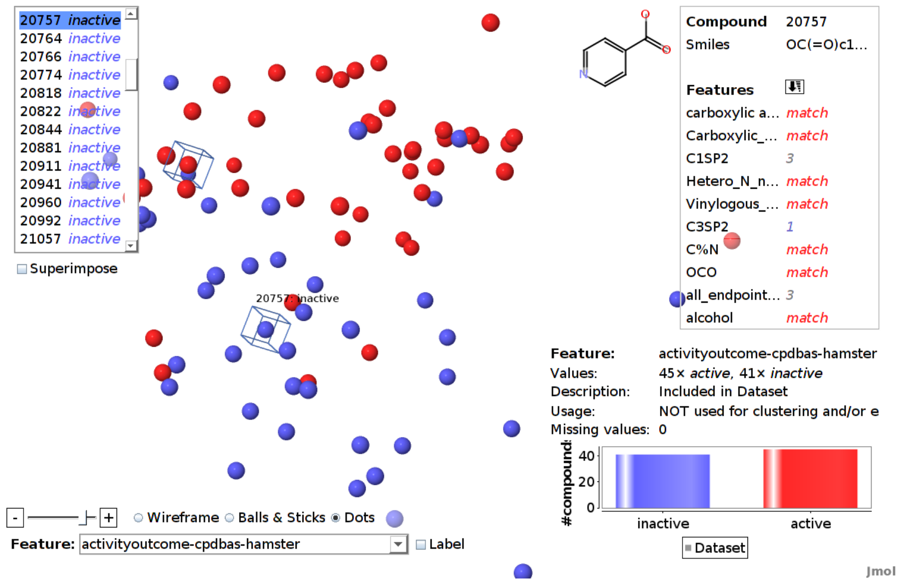

Figure 2 Applying PC and structural features to embed the CPDB hamster dataset.
Compound 20757 (*inactive*) is selected, compound 20517 (*active*) is also marked with bounding boxes. The actual activity values are highlighted. (Dots are drawn instead of compound structures to illustrate the distribution of class values and the selection of compounds.)

that have at least one carboxyl group. All 6 carboxylic acids are inactive for this endpoint, which indicates why this compound is classified correctly when taking structural fragments into account. Moreover, we can selected the nearest neighbors of the compound and inspect what they have in common. The 5 nearest neighbors are inactive, and moreover, the compound *Isonicotinic acid* and its 4 nearest neighbors are all vinylogous esters. The corresponding SMARTS fragment for vinylogous esters is matched by 12 compounds in the dataset, 10 of them are being inactive.

The mixture *1,2-Dimethylhydrazine, 2HCl* (DSSTox-RID 20517, selected in Figure 1) has the endpoint value active. It is always correctly classified by *(Q)SAR-2*, but misclassified 3 out of 5 times without structural fragments as features. Again, we gain insights when inspecting the most meaningful features for this compound separately, and for the compound including its neighbors. In fact, the compound, and its 3 nearest neighbors, belong to a group of 6 compounds that contain the fragment hydrazine (two connected aliphatic Nitrogen atoms). 5 of this 6 compounds have an active endpoint value in this dataset.

Author details
[1] Institute for Physics, Albert-Ludwigs-Universität Freiburg, Hermann Herder Str. 3, D-79104, Freiburg, Germany. [2] Information Systems, Institut für Informatik, Johannes Gutenberg - Universität Mainz, Staudingerweg 9, D-55128, Mainz, Germany.

References
1. Gold, L.S., Manley, N.B., Slone, T.H., Rohrbach, L.: Supplement to the carcinogenic potency database (CPDB): results of animal bioassays published in the general literature in 1993 to 1994 and by the national toxicology program in 1995 to 1996. Environmental Health Perspectives **107**(Suppl 4), 527–600 (1999)
