# Supplementary material for: CheS-Mapper 2.0 for visual validation of (Q)SAR models
Source: J Cheminform. 2014 Sep 23;6:41. doi: 10.1186/s13321-014-0041-7 (PMC4186979; doi:10.1186/s13321-014-0041-7)
Supplement: Additional file 3 — A KNIME Workflow for visually validating LOO-CV. A description of the KNIME workflow that is used to visually compare a leave-one-out cross-validation of the Caco-2 data. [file s13321-014-0041-7-S3.pdf]

SOFTWARE

# Additional File 3 — A KNIME Workflow for Visually Validating LOO-CV

Martin Gütlein[1], Andreas Karwath[2] and Stefan Kramer[2*]

*Correspondence: kramer@informatik.uni-mainz.de
[2] Information Systems, Institut für Informatik, Johannes Gutenberg - Universität Mainz, Staudingerweg 9, D-55128, Mainz, Germany
Full list of author information is available at the end of the article

Additional file for the article "CheS-Mapper 2.0 for Visual Validation of (Q)SAR models"

This file describes how KNIME is used to visually validate regression approaches for Caco-2 permeation [1] (see Results section of the article for description of the entire use case). We apply two different (Q)SAR approaches to model the numeric endpoint. Instead of adopting the training test split that was used in the original article, we apply a leave-one-out cross-validation procedure to compare support vector regression and simple linear regression. The visual validation workflow is implemented with the CheS-Mapper extension for KNIME. The corresponding workflow is shown in Figure 1, and can be distinguished into 3 main steps:

**Read dataset** The data is loaded into the system and data columns that are not used for modeling are filtered.

**Perform cross-validation** Two identical cross-validations are performed. The compared learning schemes are support vector machines (*SMOreg*) and linear regression, both from KNIME's WEKA extension, using default settings. Analogous to the original publication, we use the four molecular descriptors as input features: experimental distribution coefficient (*logD*), high charged polar surface area (*HCPSA*), radius of gyration (*rgyr*), and fraction of rotatable bonds (*fROTB*). The node *Numeric Scorer* computes the statistical predictivity of both models.

**Join data for CheS-Mapper** Before transferring the data into the visualization node, the modeling results are joined with each other and with the previously removed columns. Also, the prediction errors per compound, as well as the difference between both errors are computed (using the *Math Formula* nodes).

**Author details**
[1] Institute for Physics, Albert-Ludwigs-Universität Freiburg, Hermann Herder Str. 3, D-79104, Freiburg, Germany. [2] Information Systems, Institut für Informatik, Johannes Gutenberg - Universität Mainz, Staudingerweg 9, D-55128, Mainz, Germany.

**References**

1. Hou, T.J., Zhang, W., Xia, K., Qiao, X.B., Xu, X.J.: Adme evaluation in drug discovery. 5. correlation of caco-2 permeation with simple molecular properties. Journal of Chemical Information and Computer Sciences **44**(5), 1585–1600 (2004). doi:10.1021/ci049884m

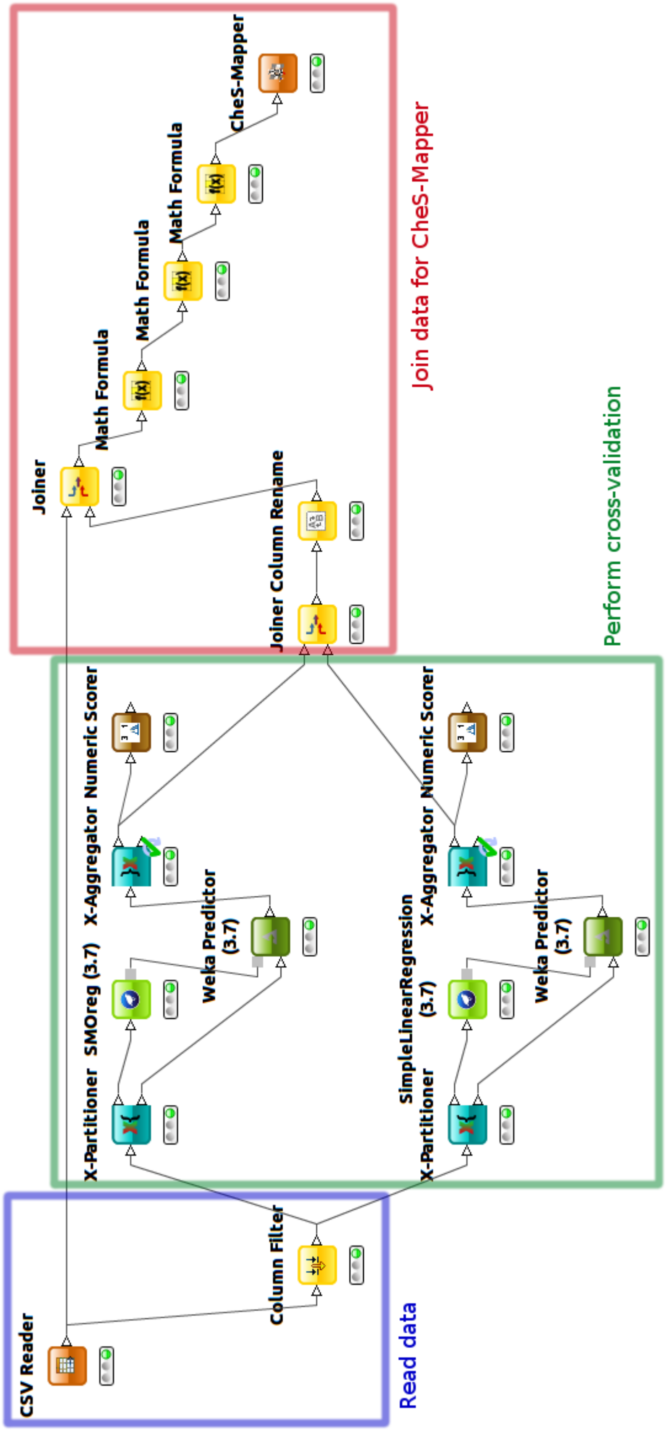


**Figure 1** Employing KNIME for cross-validation and visual validation of (Q)SARs.
This KNIME workflow employs CheS-Mapper to visually validate two regression approaches (support vector regression and simple linear regression) that are validated with leave-one-out cross-validation.
